# Motivation for sports participation, injury prevention expectations, injury risk perceptions and health problems in youth floorball players

**DOI:** 10.1007/s00167-019-05501-7

**Published:** 2019-04-13

**Authors:** Nirmala Kanthi Panagodage Perera, Ida Åkerlund, Martin Hägglund

**Affiliations:** Division of Physiotherapy, Department of Medical and Health Science, Linköping University, Linköping, Sweden

**Keywords:** Youth sports, Sports injuries, Sports participation, Injury prevention, Floorball, Youth athletes

## Abstract

**Purpose:**

Describe the motivation for floorball participation, injury prevention expectations, injury risk perceptions and prevalence of health problems in youth floorball players at the start of the season.

**Methods:**

This cross-sectional survey is part of a larger Sport Without Injury ProgrammE (SWIPE) project and provides baseline data before a cluster randomised controlled trial of an injury prevention program (Knee Control). A baseline survey (online and paper based) was collected from 47 teams with 471 youth floorball players from two provinces of Sweden before the start of the 2017 season.

**Results:**

The mean age for 140 females and 331 males was 13.7 (± 1.5) and 13.3 (± 1.0) years, respectively. The two most significant motivators for floorball participation were being part of the team (82% females, 75% males) and friends (65% females, 70% males). Fractures (84% females, 90% males), eye injuries (90% females, 83% males) and concussion (82% females, 83% males) were perceived as the most severe injuries. 93% of players believed that sports injuries can be prevented, while 74% believed it is unlikely that they will sustain an injury. Existing health problems at the beginning of the season were prevalent in 33% of players, with 65% being injuries and 35% illnesses. 17% of existing injuries at the start of the season caused time-loss from play and 17% required medical attention.

**Conclusion:**

Social aspects were the greatest motivators for floorball participation in youths, suggesting that these factors are important to retain sports participants. The high number of health problems in youth is a concern; as such more effort, resources and priority should be given to sports safety programs. Many players believed that sports injuries can be prevented, possibly providing a fertile ground for implementation of such programs.

**Level of evidence:**

IV.

## Introduction

The popularity of floorball is increasing globally, and the International Floorball Federation, which is a full member of the International Olympic Committee, has 68 member countries [11]. Floorball is one of the most popular sports in Sweden, with approximately 124,000 licensed players (81% are under 19 years old, and of these, 24% are female) registered with the Swedish Floorball Federation [11]. Floorball is a non-contact sport played indoors with a light hollow plastic ball and a graphite compound stick. A typical game involves three 20-min periods with two 10-min intermissions, though children and youth often play three 15-min periods. Each team is composed of five players and a goalkeeper. Similar to other pivoting sports, floorball involves sudden acceleration/deceleration, stops, and sharp changes of direction on a hard-playing surface and therefore involves a high risk of injury to the lower limb [18, 22]. For example, at the elite level, most injuries involve joints or ligaments, with predominantly ankle and knee injuries, including anterior cruciate ligament injuries [18, 22]. The incidence of non-contact injuries to the lower limb was reduced when using a neuromuscular training programme in adult elite female floorball players [19].

Despite the increased popularity of floorball in youth athletes, much of the available research regarding injuries in floorball focuses on elite athletes or adults. Injury panorama and injury risk factors and injury mechanisms may be different between youth and adults owing to the physiological differences between these groups, including muscle function, physical development, and differences in joint kinematics, skill levels and workloads. As such, the available evidence relating to adult elite athletes cannot necessarily be extended to youth players.

The risk of injury may also impact sports participation. Sports and sporting clubs provide opportunities and settings for people to be active and live a healthy lifestyle; this is particularly important given that inactivity is a significant current public health concern [4, 9]. Close social relationships (particularly parents and peer relationships), friendship quality, fitness and health, competence, sports events, enjoyment and relaxation through sports have been identified as motivators for youth athletes to continue participating in sports [12, 23]. However, the motivation for sports participation significantly varied between sexes, where popularity was a stronger motivation for sports participation in males than females [12]. Currently, no study has investigated motivators for participation in floorball among youth players. Such information is important for the governing sport bodies in order to embed strategies to sustain participants. This study aims to be the first to investigate motivation for floorball participation, injury prevention expectations, injury risk perceptions and prevalence of health problems in youth floorball players at the start of the season.

## Materials and methods

**Fig. 1:**
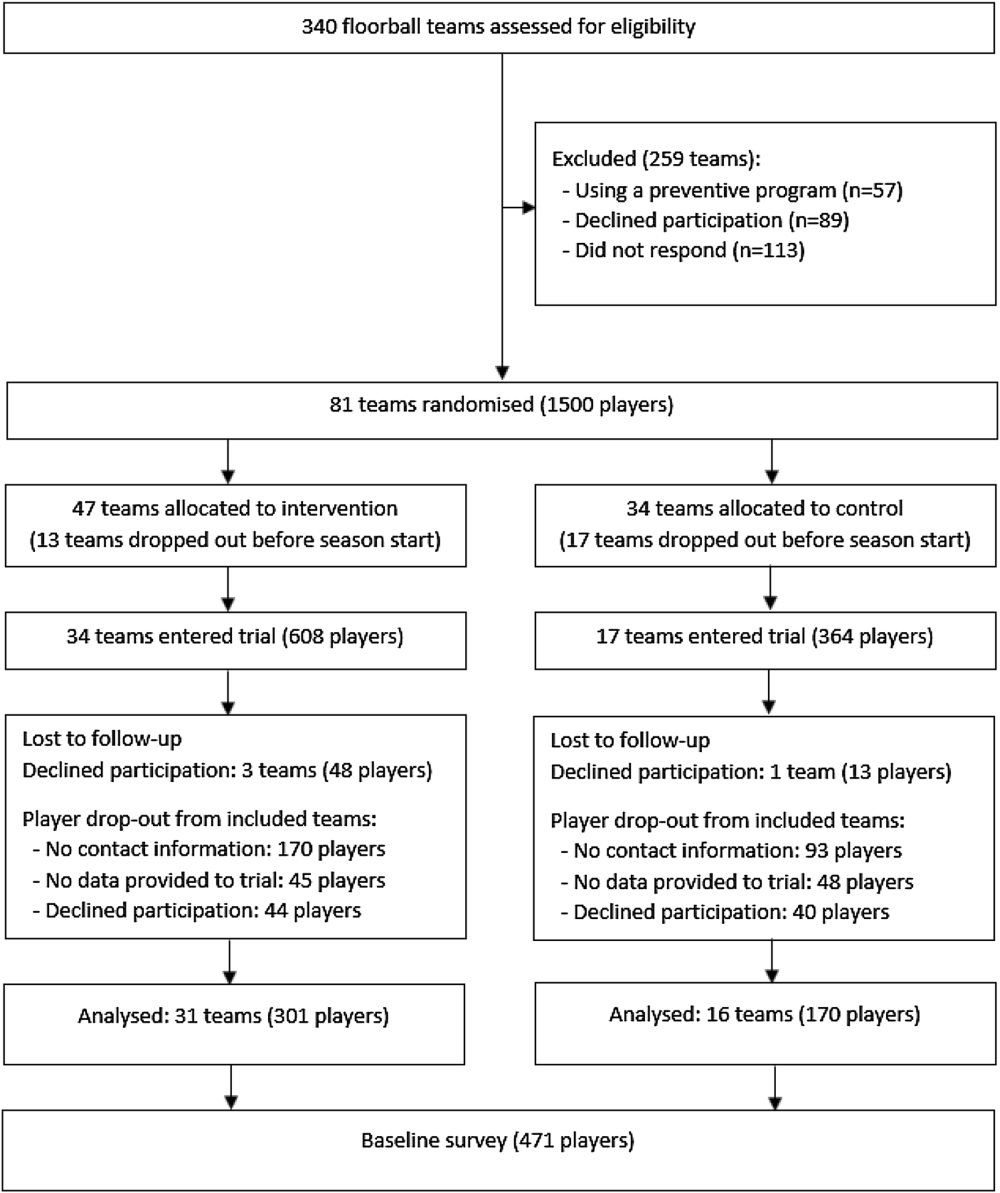
Study participant recruitment for the cluster randomised controlled trial of an injury prevention program (Knee Control) in youth floorball

This cross-sectional survey is part of a larger Sport Without Injury ProgrammE (SWIPE) project and provides baseline data before a cluster randomised controlled efficacy trial of an injury prevention program (Knee Control). Inclusion criteria were youth floorball players aged 12–17 years who were registered to play floorball at a club in two provinces of Southern Sweden (Östergötland and Småland) for the 2017–2018 season. Included teams should not have used the Knee Control injury prevention exercise program, or similar structured injury prevention exercise program, in the last year; and should train at least twice a week to obtain an adequate dose of the training intervention. Study participant recruitment is illustrated in Fig. 1. A survey was distributed in paper-based and online forms to all participating youth floorball teams before the start of the season in October 2017. Two reminders were sent to non-responders (players) and one reminder to coaches of these teams via email and SMS.

The baseline survey included 20 questions relating to player demographic characteristics, sports participation, injury prevention expectations and injury risk perceptions (Table 1). For example, a seven-point Likert scale was used for the question relating to motivation for sports participation. For the analysis responses of 1–3 were grouped as low significance, 4 was considered neutral and 5–7 were grouped as high significance. The same seven-point Likert scale and grouping strategy was used for questions relating to perception of injury risk and injury severity.

**Table 1:** Sport Without Injury ProgrammE (SWIPE) baseline survey questions used to collect motivation for floorball participation, health problems and injury prevention expectations and injury risk perceptions data at the baseline

Motivation for floorball participation
How significantly have the following factors impacted your interest and motivation to participate in floorball? Mother, father, sibling, friends, coach, team, club environment, proximity to the training facility, your sporting success (1 low significance–7 high significance)
Injury prevention expectations and injury risk perceptions
Do you use floorball protective eyewear (goggles)? (Yes, always for practice and games; Only for practice; Only for match; Sometimes; Never)
Have you used the Knee Control program (or similar program) before? The Knee Control program is a training program consisting of six different exercises: one-legged squat, pelvic lift, two-legged squat, the bench, lunges, jump and landing (Yes, regularly in the past year; Yes, on and off in the past year; No; Do not know)
In your opinion, how serious are the following types of injuries? Ankle sprains, knee sprains, muscle strains, fractures, lacerations, bruises, concussions, eye injuries, dental injuries (1 not serious–7 very serious)
I believe I will get injured during this season (1 extremely unlikely–7 extremely likely)
Many sports injuries can be avoided with training or protective equipment (1 false–7 true)
Health problems
How stressed have you felt in the past week? (0 not stressed–10 very stressed)
How did you sleep in the past week? (0 very good–10 very bad)
How did you feel in the past week? (0 very good–10 very bad)
Have you had any difficulties participating in normal floorball training during the past week due to health problems (such as pain, aches, tenderness, stiffness), injury or illness?^a^ (0–Full participation without health problems, injury or illness; 8–Full participation, but with health problems, injury or illness; 17–Reduced participation due to health problems, injury or illness; 25–Cannot participate due to health problems, injury or illness)
To what extent have you reduced your training volume due to health problems/injury during the past week?^b^ (0–No reduction; 6–To a minor extent; 13–To a moderate extent; 19–To a major extent; 25–Cannot participate at all)
To what extent has your health problem/injury affected your performance during the past week?^b^ (0–No effect; 8–To a minor extent; 13–To a moderate extent; 19–To a major extent; 25–Cannot participate at all)
To what extent have you experienced pain related to floorball during the past week?^b^ (0–No pain; 8–Mild pain; 17–Moderate pain; 25–Severe pain; 25–Cannot participate at all*)

^a^Question from the Oslo Sports Trauma Research Center questionnaire on health problems [7]

^b^Questions from the Oslo Sports Trauma Research Center overuse injury questionnaire [6]

*Added as an extra option to original answer options

To collect baseline health problem data, all players completed the Oslo Sports Trauma Research Center (OSTRC) questionnaire on health problems [7] and overuse injury questionnaire [6] at the beginning of the season. Each question response is marked with between 0 and 25 points where 0 represented no health problem and 25 was a severe problem (Table 1). The scores were summed to calculate the OSTRC severity score (range 0–100), and a score of ≥ 40 was considered as a severe health problem.

This study was approved by the Regional Ethical Review Board in Linköping (Project number Dnr 2017/294-31).

### Statistical analysis

Data were analysed using SPSS^®^ 25.0 (IBM SPSS Statistics 2015), and descriptive statistics were used to motivation for floorball participation, injury prevention expectations, injury risk perception and reported health problems. As the data were non-normally distributed, a Mann–Whitney *U* test was conducted to compare differences between two independent groups. A *P* value of ≤ 0.05 was considered significant.

## Results

### Survey respondents’ characteristics

In total, 471 players were included in this pre-season survey. The study participants (Table 2) were 140 females (mean age 13.7 ± 1.5 years) and 331 males (mean age 13.3 ± 1.0 years). Of the female participants, 62% (*n* = 93) had menarche. On average, the youth had played floorball for 4.9 ± 2.3 years. Most youth (65%, *n* = 291) also played other sports, and a majority of these (76%, *n* = 218) played football. Also, 17% (*n* = 80) of the youth attended a school with sports specialisation, and 7% (*n* = 34) were at a school with floorball specialisation. Most youth (51% of females and 55% of males) trained and played floorball three times per week in their teams, and the majority of players (69% of females and 76% of males) perceived their training volume to be high.

**Table 2:** Demographic characteristics and self-reported training frequency at baseline for female (*n* = 140) and male (*n* = 331) youth floorball players

	Female (%)	Male (%)
Age		
Mean	13.7 ± 1.5	13.3 ± 1.0
Median	14	13
12	28.5	26.3
13	19.7	28.4
14	25.5	36.9
15	6.6	4.8
≥ 16	19.4	3.6
Mean no of years of floorball experience	4.5 ± 2.5	5.1 ± 2.3
No of times per week floorball played in the team (training/match)		
1 time/week	2.2	1.9
2 times/week	2.2	16.4
3 times/week	51.1	55.0
4 times/week	16.1	19.8
≥ 5 times/week	9.5	6.9
No of times per week floorball training at school		
Not applicable	92.5	84.9
1 time/week	3.0	5.0
2 times/week	3.0	8.5
≥ 3 times/week	1.5	1.5
No of times per week trained and played other sports		
Not applicable	42.5	35.0
1 time/week	18.1	15.3
2 times/week	11.0	24.0
3 times/week	18.1	12.7
4 times/week	6.3	9.0
≥ 5 times/week	3.9	4.0
Perceived current training volume per week		
Extremely low	0.8	0.3
Very low	4.5	1.3
Fairly low	21.2	5.4
Neither high or low	40.9	13.6
Fairly high	31.8	37.5
Very high	0.8	39.4
Extremely high	0.8	2.5

### Motivation for floorball participation

**Fig. 2:**
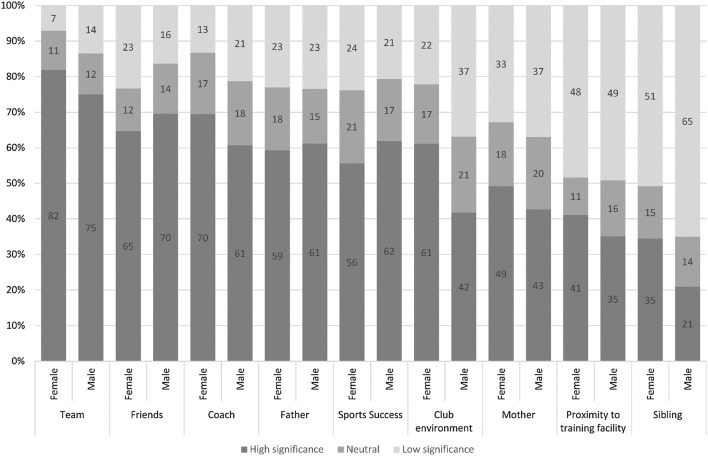
Factors motivating youth to play floorball (*n* = 471)

Motivation from the father to play floorball was significant for both male and female youth; however, the team was the greatest motivation for floorball participation in all players (Fig. 2). Siblings, coach, club environment and proximity to the facility were also significant motivators for floorball participation in females. For male floorball players, friends and sporting success were significant motivators.

### Injury prevention expectations and injury risk perceptions

Protective glasses were worn by 85% of the players during training and match play, with 88% of male and 78% of female players always using protective eyewear. However, 9% never used protective glasses, with more female players (15%) not using protective eyewear than males (7%). Protective glasses must be worn by players up until 15 years of age at matches; however, only 87% (*n* = 352) of players aged under 15 years always wore protective glasses in training and matches and 4% (*n* = 16) of players this age never wore protective eyewear. Of players over 15 years, 45% (*n* = 26) never wore protective glasses, and another 45% (*n* = 26) always wore protective glasses both at training and at matches.

The Knee Control injury prevention exercise program had been used regularly by 14% (17% females and 12% males) of players and it was used sometimes by 28% (39% females, 27% males) players when they participated in other sports during the past year. Of all the players, 47% (37% females, 51% males) had never used the Knee Control program.

**Fig. 3:**
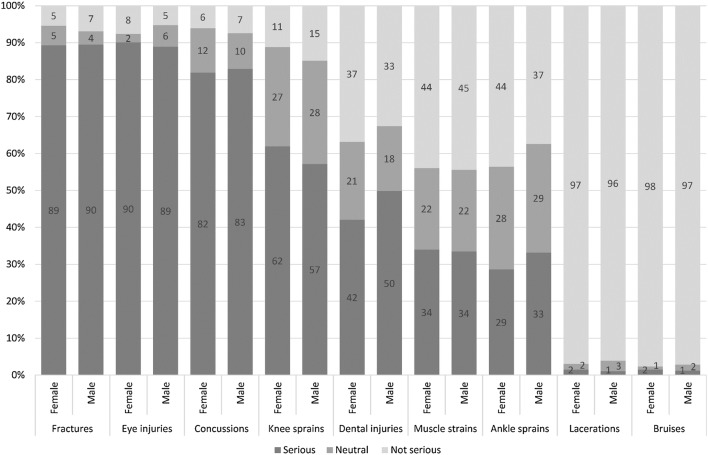
Overview of the self-reported perception of injury severity at the beginning of the season (*n* = 471)

Of the potential injuries that can be sustained in floorball, fractures, eye injuries and concussions were perceived to be the most severe injuries, with lacerations and bruises perceived to be less severe (Fig. 3). There were no distinct sex differences in perception of the severity of injuries.

When the players were asked about their perception of likelihood of sustaining an injury during the season, 42% (43% females and 41% males) thought themselves unlikely to sustain an injury and 32% (31% females and 33% males) were neutral and believed an injury to be neither likely nor unlikely. In contrast, 26% of both females and males saw a likelihood that they could be injured playing floorball. Most players (93%) believed that sports injuries can be prevented, and only 3% did not believe that sports injuries can be prevented. There were no sex differences in the perceptions of injury risk or the possibility of preventing sports injuries.

### Health problems at the start of the season

**Fig. 4:**
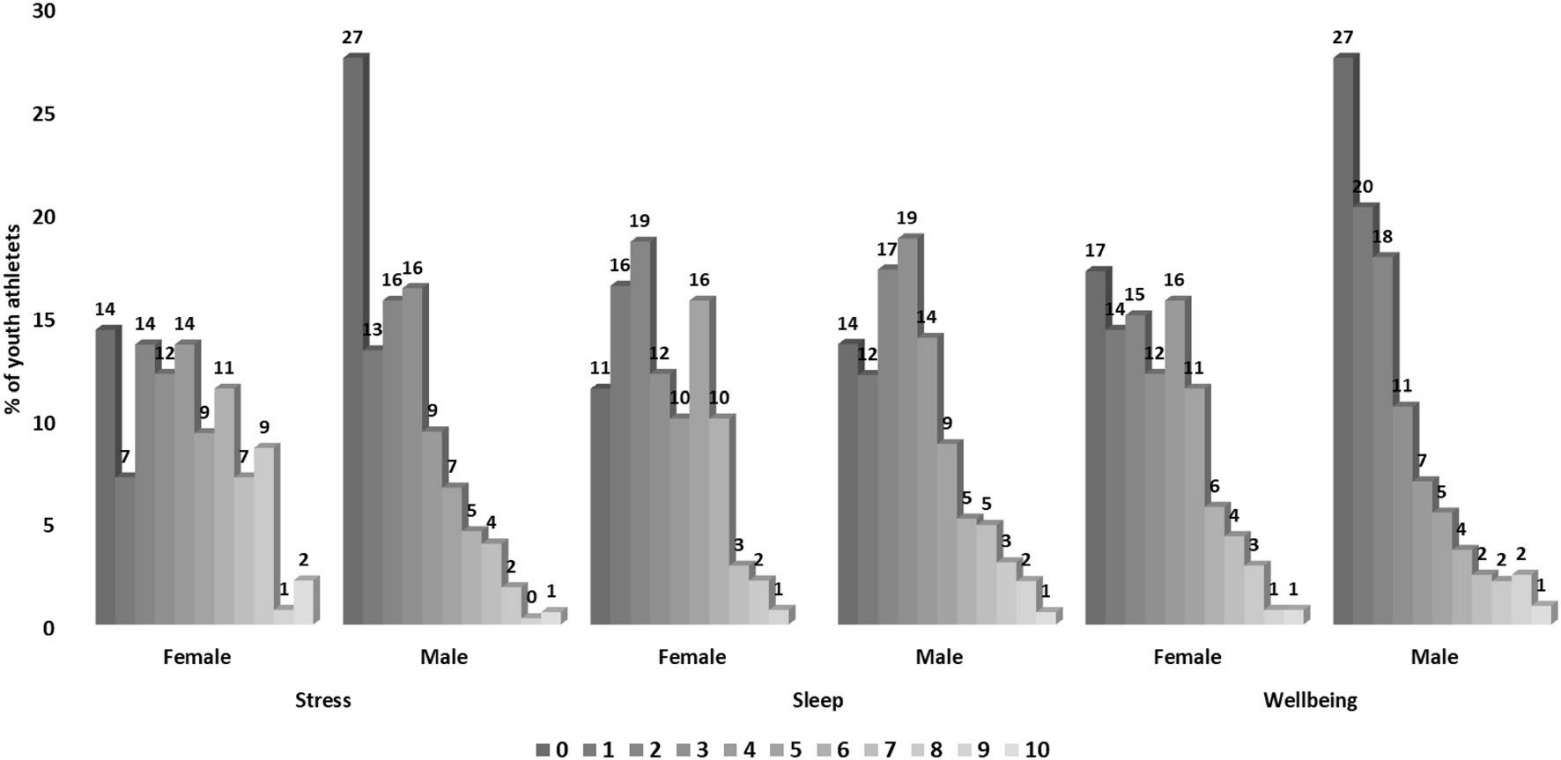
Self-reported stress, sleep and well-being at the beginning of the season. Scale: Stress 0 (not stressed) to 10 (very stressed); Sleep 0 (very good) to 10 (very bad); Well-being 0 (very good) to 10 (very bad

At the start of the season, most of the players reported good sleep and well-being (Fig. 4). On a scale of 0 (not stressed) to 10 (very stressed), the youth floorball players reported a mean stress of 2.8 ± 2.5 at the start of the season. On a scale of 0 (very good) to 10 (very bad), players reported mean sleep of 3.1 ± 2.3, and well-being of 2.5 ± 2.4. Youth female floorball players felt more stress with a median of 4 (IQR 2–6) compared to males with a median of 2 (IQR 0–4, *P *= 0.000). Contrary, females reported better well-being than males with median of 3 (IQR 1–5) versus 2 (IQR 0–3, *P* = 0.000). There was no difference in sleep between females, median 3 (IQR 1–5) and males, median 3 (IQR 0–3, n.s.).

Question 1 of the OSTRC questionnaire on health problems indicated that 33% (*n* = 148, 38% females and 30% males) of youth players were unable to fully participate in the sport without any health problems at the start of the floorball season. The reported health problems were 64% (*n* = 95) injuries and 36% (*n* = 53) illnesses. Of all injuries, 17% (13% females and 19% males) caused time-loss from sports and 17% (33% females and 9% males) required medical attention. Injuries were mainly to the lower limb (total 70%, 64% females, 72% males) and primarily located to the knee (total 48%, 50% females, 47% males).

Of all players, 85% (84% females, 87% males) played floorball (training or match) the week of the baseline survey. As illustrated in Table 3, 81% (77% females, 83% males) of players reported no reduction in their training volume at the start of the season. Pain was reported by 28% (32% females, 26% males) of players, and 13% of female and 8% of male players could not participate in floorball due to pain. Of the 95 reported injuries at the start of the season 28% (20% females and 31% males) were acute injuries and 72% (80% females and 69% males) were overuse injuries.

**Table 3:** Overview of the responses to the OSTRC questionnaire on health problems

	Female *n* = 140 (%)	Male *n* = 331 (%)
OSTRC Health^a^ Question 1 (participation in normal training)		
Full participation without health problems, injury or illness	61.7	70.1
Full participation, but with health problems, injury or illness	18.8	15.3
Reduced participation due to health problems, injury or illness	7.5	6.8
Cannot participate due to health problems, injury or illness	12.0	7.8
OSTRC Injury^b^ Question 2 (reduced training volume)		
No reduction	76.7	83.3
To a minor extent	7.5	6.1
To a moderate extent	1.7	1.4
To a major extent	0.8	1.0
Cannot participate at all	13.3	8.2
OSTRC Injury^b^ Question 3 (effect on performance)		
No effect	70.8	75.7
To a minor extent	9.2	10.6
To a moderate extent	5.8	4.1
To a major extent	1.7	1.4
Cannot participate at all	12.5	8.2
OSTRC Injury^b^ Question 4 (pain)		
No pain	68.3	74.0
Mild pain	10.8	9.2
Moderate pain	6.7	7.2
Severe pain	1.7	1.4
Cannot participate at all*	12.5	8.2

^a^Question 1 from the Oslo Sports Trauma Research Center questionnaire on health problems [7]

^b^Questions 2–4 from the Oslo Sports Trauma Research Center overuse injury questionnaire [6]

*Added as an extra option to original answer options

When the OSTRC severity score (range 0–100) was calculated, the mean severity score was 15 ± 30, with 13.6% (*n* = 61) of players reporting severe health problems (score ≥ 40) at the beginning of the season. There were more severe health problems in female floorball players (17%) than males (12%), whilst this did not reach statistical significance (*U *= 18,631.5, n.s.). Youth with health problems demonstrated significantly more stress, poorer sleep quality and poorer well-being than youth that reported no health problem at the start of the season (Table 4).

**Table 4:** The relationship between health problems, stress, sleep, well-being, training volume and importance of sporting success at the beginning of the season

	Youth with health problem *n* = 148	Youth without health problem *n* = 301	*P* value
Stress (not stressed 0–10 very stressed)	3 (2–6)	2 (0–4)	0.001
Sleep (very good 0–10 very bad)	3 (2–5)	3 (1–4)	0.001
Well-being (very good 0–10 very bad)	3 (1–5)	2 (0–3)	0.000
Weekly training volume	5 (5–6)	5 (5–6)	n.s.
Motivation from sporting success	5 (4–6)	5 (4–6)	n.s.

Values are median (IQR)

## Discussion

The most important finding of the present study was that one in three players reported a health problem at the start of the season providing insight into the status of the athletes’ health leading into the season. The high prevalence of health problems is a concern; if injuries are left untreated, there is a potential for more severe and long-term adverse health consequences [3, 5, 15, 27, 28]. A novel finding of this study is that key motivators for floorball participation in youths were identified, providing important information to the sporting organisations aiming to retain sports participants.

### Motivation for floorball participation

Understanding the factors that motivate for sports participation is particularly important when many countries, including Sweden, are attempting to increase activity levels in adolescents through sustainable youth sports programs. As the umbrella organisation for approximately 70 federations, the Swedish Sports Confederation’s strategy for Swedish sport 2025 has a strong focus on getting as many people as possible to play sports for as long as possible [20]. Our study found that a young player’s father, friends, coach and team significantly motivated them to participate in floorball. This might suggest that most youths are active in floorball because of the social aspects, i.e. that it is fun and allows them to be with their friends and be part of a team, and this should be a focus in youth sports to retain sports participants.

### Overview of health problems and injury prevention expectations

The finding of this study revealed that 33% of non-elite youth floorball players reported a health problem at the beginning of the season extends previous research in Swedish elite youth athletes showing that one in three suffer an injury every week [25]. Similarly one in four Australian youth athletes participating in multiple contact sports reported an injury [10]. This is concerning, and it is likely that many athletes in our study carried an injury/health problem from the summer season that affected them at the start of the floorball season. The summer and winter sports seasons overlap with no clear pre-season and, therefore, players who play multiple sports may transition from one to other with little or no rest in-between. Although there are some similarities between pivoting sports such as floorball and football, it can be argued that the different surfaces (indoor vs. outdoor) and type of play can change the type of load [21]. Further, without a pre-season, athletes do not have adequate time to prepare for the sport with specific drills, thereby possibly increasing the risk of injury. Increasing adequate pre-season preparation can potentially reduce the risk of injury [13, 14, 26].

Research into the incidence and prevalence of overuse injuries in youth sports is scarce [8] and use of time-loss injury definitions underestimate the actual magnitude of overuse injuries [6]. We found that 28% of youth floorball players reported pain or were unable to play due to pain, with approximately three out of four injuries being overuse-related. A six-month prospective cohort study of Norwegian elite youth athletes playing a variety of endurance, team, and technical sports reported similar findings, with 37% players reporting overuse injuries [17]. The large number of overuse injuries is of concern in these young athletes. Youth specialising in one sport at an early age and training year-round have increased risk of overuse injuries [8]. This may be due to the structural damage and weakness because of inadequate recovery time and excessive stress impacting tissue remodelling. 17% of the youth included in this study attended a school with a sports profile, and this might be another plausible explanation to the high prevalence of health problems reported in this study. Playing a variety of different sports throughout the year as a form of cross-training may benefit physical development as well as develop transferable skills.

A majority of the youth floorball players felt that their training volume was high or very high. In youth athletes playing multiple contact team sports, the relationship between risk of injury and training volume is linear [10]. In addition, poor sleep, poor well-being and high stress have been associated with health problems [8, 24] and are modifiable injury risk factors. Therefore, it is essential to consider the impact of training load and recovery, particularly in youth athletes who have a developing musculoskeletal system. The preliminary cross-sectional findings of this study from the start of the season should be verified further using prospectively recorded data from the full season.

In this study cohort, most of the same athletes play football in the spring to autumn and then floorball in the winter to spring, and many of their parental coaches also transition between sports. Therefore, it is economical and practical to implement an injury prevention exercise program such as Knee Control for both sports, so the athletes can maintain their prevention program when they transition between sports. The larger SWIPE study excluded the floorball teams that already used the Knee Control injury prevention program, but 42% of the players still had used it in the last year as part of the other sport they play. This consistency in their preventive training is likely to be beneficial. Approximately 93% of the youth floorball players in this study considered that injuries could be prevented, indicating that there is reasonable ground to implement injury prevention programs.

### Injury risk perceptions

Although there were no concussions reported at the beginning of the floorball season, most of the players perceived concussion to be a severe injury, indicating good awareness. This may be because of increased discussions, education campaigns and awareness programs of the importance of concussion management in sports. Also, the majority perceived eye injuries as serious, likely due to the increased attention on eye injuries in floorball. In Sweden, it is mandatory to wear protective eyewear until age 15 at floorball matches. Interestingly, 9% of the youth in our study never used protective eyewear, and 2% used them sometimes. Also, only 87% of players under 15 years always used protective eyewear, indicating that players are not fully adhering to mandatory safety requirements during matches and safety recommendations for training. It is possible that these players were playing matches with older peers where the protective eyewear rule was not as strictly enforced. It is also worrisome that 45% of players above 15 years never wore protective eyewear, since floorball is a high-risk sport for eye injuries, potentially leading to severe and enduring problems [2]. Four of ten players thought they were unlikely to sustain an injury during the floorball season and another 32% were neutral about a potential injury. More education and discussions surrounding injuries and prevention strategies, and to develop and make available easily accessible resources for the floorball players as part of sports safety initiative to promote safe and healthy sports is needed.

The use of single item global health questions to gain information about stress, sleep and well-being of the youth athletes can be a limitation of this study. Single item global health questions are established to provide reliable and valid information and are widely used in population health surveys, but they might not provide comprehensive and detailed information that can be gleaned from a multi-item measurement scale [1]. However, youths may find it difficult to perceive nuances in similarly alike questions in a multi-item survey and thus may answer all the questions in the same manner. Therefore, single item global health questions are deemed adequate to gain a “snap shot” of baseline general health status (stress, sleep and well-being) of youth athletes and the simplicity of these questions minimise the burden on study participants as they are taking part in a lengthy RCT. Another limitation is that the survey instrument was not validated for test–retest in youth floorball players. However, most of the questions used in this baseline survey were adopted from previous studies [6, 7, 16] and some of the content was modified to ensure that the survey is relevant and specific for youth floorball players in order to collect accurate self-reported data. Finally, due to the self-reported nature of data collection and reliance on young athletes’ memory, recall bias can be another limitation of this study. Prompting questions and limiting recall period to 1 week was used to overcome this limitation.

## Conclusions

Social aspects were the greatest motivators for floorball participation, and organisations that aim to grow sports should focus more on this to retain participants. The high prevalence of health problems in youth at the beginning of a season is a concern; as such more effort, resources and priority should be given to sports safety programs. Many players believed that sports injuries can be prevented, possibly providing a fertile ground for implementation of such programs.

## Abbreviations

SWIPE: Sport Without Injury ProgrammE
OSTRC: Oslo Sports Trauma Research Center
